# Macrolide and fluoroquinolone resistance-associated mutations in *Mycoplasma genitalium* in Johannesburg, South Africa, 2007–2014

**DOI:** 10.1186/s12879-019-3797-6

**Published:** 2019-02-13

**Authors:** Etienne E. Muller, Mahlape P. Mahlangu, David A. Lewis, Ranmini S. Kularatne

**Affiliations:** 1 Sexually Transmitted Infections Section, Centre for HIV and Sexually Transmitted Infections, National Institute for Communicable Diseases, National Health Laboratory Service, Johannesburg, South Africa; 2 Western Sydney Sexual Health Centre, Western Sydney Local Health District, Parramatta, Australia; 3 Marie Bashir Institute for Infectious Diseases and Biosecurity and Sydney Medical School-Westmead, University of Sydney, Sydney, Australia; 4 Department of Clinical Microbiology and Infectious Diseases, Faculty of Health Sciences, University of the Witwatersrand, Johannesburg, South Africa

**Keywords:** Macrolide, Fluoroquinolone, Resistance-associated mutations, *Mycoplasma genitalium*, Johannesburg, South Africa

## Abstract

**Background:**

Antimicrobial resistance in *Mycoplasma genitalium* is rising globally with resultant clinical treatment failure. We investigated the prevalence of mutations in the macrolide and fluoroquinolone resistance-determining regions of *M. genitalium* in Johannesburg, South Africa, and ascertained their association with HIV serostatus.

**Methods:**

Stored *M. genitalium* positive specimens, collected from STI and HIV patients enrolled in the Gauteng STI National Microbiological Surveillance programme (2007–2014) and a large HIV outpatient clinic-based study (2007) in Johannesburg, were tested for antimicrobial resistance.

**Results:**

We determined the prevalence of 23S rRNA gene mutations conferring macrolide resistance and mutations in the quinolone resistance-determining regions (QRDR) of the *gyrA* and *parC* genes in 266 *M. genitalium* positive DNA extracts. No macrolide resistance-associated mutations were detected in any of the specimens analysed. QRDR mutations with known *M. genitalium*-associated fluoroquinolone resistance were not detected in *gyrA*, however, one specimen (0.4%) contained a D87Y amino acid alteration in *parC,* which has been linked to fluoroquinolone treatment failure. The most common *parC* amino acid change detected, of unknown clinical significance, was P62S (18.8%). We found no significant association between QRDR mutations in *M. genitalium* and HIV-infection.

**Conclusions:**

Ongoing antimicrobial resistance surveillance in *M. genitalium* is essential, as macrolide resistance may emerge given the recent incorporation of azithromycin into the 2015 South African national STI syndromic management guidelines.

## Background

*Mycoplasma genitalium* is a known cause of non-gonococcal urethritis (NGU) in men and cervicitis in women [1]. A recent meta-analysis revealed that *M. genitalium* infection in women is also associated with an increased risk of pelvic inflammatory disease (PID), infertility, preterm delivery and spontaneous abortion [2]. Furthermore, *M. genitalium* infection is a possible cofactor for the acquisition and transmission of human immunodeficiency virus (HIV) [3–5]. The prevalence of *M. genitalium* infection ranges between 1.1 and 3.3% in the general population and can be as high as 35% among men with symptomatic non-chlamydial NGU [1, 6]. Asymptomatic *M. genitalium* infections are common among women and often remain undiagnosed [7].

The current STI Management Guidelines (2015) for South Africa include the use of 1 g azithromycin (a macrolide), orally, as a single dose for the treatment of male urethritis syndrome (MUS) and vaginal discharge syndrome (VDS) [8]. The European NGU management guidelines recommend the use of an extended 5-day azithromycin regimen (500 mg on the first day and 250 mg for the next four days) for dual treatment of *M. genitalium* and *Chlamydia trachomatis* infections [9]. The widespread use of azithromycin has led to the emergence of macrolide resistance in some settings [10–12]. A rapid decline in azithromycin efficacy, from 85% in 2009 to 61% in recent years has been documented in the Asia-Pacific region [10, 13, 14]. Resistance is mediated by nucleotide substitutions at positions A2058 and A2059 (*Escherichia coli* numbering) in the V region of the 23S rRNA gene of *M. genitalium* [15]. Mutations in the L4 and L22 ribosomal protein genes conferring macrolide resistance have been described in other mollicutes but these mutations seem to be associated with low-grade resistance [16]. Moxifloxacin, a fluoroquinolone, is often recommended as a second-line treatment in cases of azithromycin treatment failure; however, reports of moxifoxacin treatment failure, have been described, especially in the Asia-Pacific region, and are associated with point mutations in the quinolone resistance-determining region (QRDR) of the DNA gyrase (*gyrA*) and the topoisomerase IV (*parC*) genes [10, 17–21]. In our setting, HIV-infected individuals, in particular, may have increased exposure to fluoroquinolones used in the treatment of acute gastroenteritis and non-typhoidal salmonella infections and macrolides for the treatment of severe pneumonia [22–25]. The isolation of *M. genitalium* in cell culture remains problematic and therefore in vitro antimicrobial susceptibility testing of *M. genitalium* strains is rarely performed [26]. The genetic characterisation of *M. genitalium* strains from South Africa is important for studies of antimicrobial resistance, which in turn enables the development of effective treatment algorithms for syndromic management of patients with genital discharge.

We evaluated the prevalence of macrolide and fluoroquinolone resistance in stored *M. genitalium* positive specimens at the National Institute for Communicable Diseases (NICD), South Africa. We sequenced the region V of the 23S rRNA gene and QRDRs of the *gyrA* and *parC* genes in *M. genitalium* positive specimens obtained from Gauteng STI National Microbiological Surveillance (NMS) (2007–2014), as well as in *M. genitalium* positive specimens obtained from HIV positive patients previously recruited to an urban HIV outpatient clinic in Johannesburg, South Africa (2007). Additionally, we sought to determine the association between HIV serostatus and the presence of mutations in the antimicrobial resistance-determining regions of *M. genitalium*.

## Methods

### Study population and specimens

The STI Section of the Centre for HIV & STI (CHIVSTI) at the NICD, Johannesburg, has conducted annual STI National Microbiological Surveillance (NMS) surveys among STI patients (male and female patients aged 18 or older) attending the Alexandra Health Centre in Gauteng, South Africa, since 2007. This facility is a community-based primary healthcare centre offering HIV, AIDS, STI and TB-related treatment, care and support services and is situated in the most populous province in South Africa. Clinical and microbiological data were linked using survey numbers and delinked from patient identifiers. Genomic DNA was initially extracted from urine (men) and endocervical swabs (women) using an automated DNA extractor (X-tractor Gene and QIAxtractor platforms, Qiagen, Hilden, Germany). Extracted DNA specimens were tested for *Neisseria gonorrhoeae*, *C. trachomatis*, *Trichomonas vaginalis* and *M. genitalium* using a validated in-house multiplex real-time PCR assay on the Rotor-Gene platform (Qiagen, Hilden, Germany) [27]. A total of 458/4731(9.7%) of Gauteng NMS specimens analysed during 2007–2014 tested positive for *M. genitalium* DNA, of which 222/2509 (8.8%) were from males and 236/2222 (10.6%) from females. These 458 *M. genitalium* positive DNA specimens were stored at -70 °C at the STI Section, CHIVSTI and were used for the study. Additional stored *M. genitalium* DNA specimens, obtained from a previous study that determined the burden of asymptomatic STIs among people living with HIV/AIDS (PLWHA) at an urban HIV outpatient clinic in Johannesburg, were also included in this study [28]. The presence of *M. genitalium* DNA in these specimens was determined using the same methodology described above. A total of 68/1108 (6.1%) of PLWHA specimens also tested positive for *M. genitalium* DNA; these positive DNA extracts were stored at -70 °C at the STI Section, CHIVSTI and used in this analysis. The total number of *M. genitalium*-positive specimens that were available for this study is depicted in Table 1. All 526 frozen stored *M. genitalium* DNA extracts were re-tested to confirm the presence of *M. genitalium* DNA using a validated, commercially available Sacace *M. genitalium* Real-TM PCR assay (Sacace Biotechnologies, Como, Italy), according to manufacturer’s instructions. The HIV serostatus of each de-identified NMS participant was determined as part of the main NMS study protocol.

**Table 1:** Overall *M. genitalium* prevalence and number of *M. genitalium* positive specimens after confirmation testing (2007–2014)

Year	Study^*^	Males					Females				
		N	*M. genitalium* prevalence		*M. genitalium* used in this study after confirmation		N	*M. genitalium* prevalence		*M. genitalium* used in this study after confirmation	
			n	%^#^	n	%^§^		n	%^#^	n	%^§^
2007	NMS	205	31	15.1	2	6.5	200	23	11.5	1	4.3
2007	PLWHA	550	39	7.1	16	41.0	558	29	5.2	8	27.6
2008	NMS	433	37	8.5	14	37.8	312	32	10.3	13	40.6
2009	NMS	499	45	9.0	13	28.9	492	40	8.1	7	17.5
2010	NMS	427	38	8.9	27	71.1	393	51	13.0	36	70.6
2011	NMS	261	21	8.0	16	76.2	220	19	6.4	15	78.9
2012	NMS	198	16	8.1	15	93.8	207	20	9.7	16	80.0
2013	NMS	280	23	8.2	13	56.5	199	27	13.6	25	92.6
2014	NMS	206	11	5.3	10	90.9	199	24	12.1	19	79.2
Total	Total	3059	261	8.5	126	48.3	2780	265	9.5	140	52.8

^*^ *NMS* National Microbiological Surveillance study (symptomatic patients), *PLWHA* People living with HIV/AIDS study (asymptomatic patients)

^#^ Percentage of genital discharge specimens that initially tested positive for *M. genitalium*

^§^ Percentage of stored DNA specimens that tested *M. genitalium* positive after confirmatory testing in this study

### Detection of mutations in the 23S rRNA gene conferring macrolide resistance and characterization of the quinolone-resistance determining regions of the *gyrA* and *parC* genes of *Mycoplasma genitalium*

Macrolide resistance mediating mutations in region V of the 23S rRNA-gene were detected by DNA sequencing of amplicons obtained from the *M. genitalium* positive specimens. Published primers targeting a unique 147 bp region of the *M. genitalium* 23S rRNA gene flanking the mutations found in region V of the 23S rRNA gene were used [15]. Fluoroquinolone resistance testing was conducted based on the methodology described by Shimada et al. (2010) [29]. Mutations in the region corresponding to the QRDR of the *E. coli* DNA gyrase genes (*gyrA*) and the topoisomerase IV (*parC*) genes in *M. genitalium* were analysed. PCR primers for the amplification of the *gyrA* and *parC* genes have been published previously [29]. The PCR assays were performed in a GeneAmp 9700 thermocycler (Applied Biosystems, Foster City, CA, USA). To visualize PCR products, 1 μl of PCR products, including the 1000 bp marker were analyzed on an Agilent 2100 Bioanalyzer (Agilent Technologies, Santa Clara, CA, USA). The PCR products were purified using the MSB® Spin PCRapace kit (Invitek, Berlin, Germany). After purification of the PCR products, sequencing reactions were performed in both directions using the same primers as previously described [15, 29]. Cycle sequencing was performed using the ABI Prism Big Dye terminator reaction kit v. 3.1 (Applied Biosystems, Foster City, CA, USA) and sequences were analysed on a 16-capillary ABI 3130xl system (Applied Biosystems).

### Data analyses

Contigs obtained from initial sequencing were assembled using Sequencher software version 5.4.6 (Gene Codes Corp, MI, USA) and the FASTA sequences obtained were aligned with reference sequences using MEGA version 7.0.20 [30]. A chi-square test was used to determine associations between HIV serostatus and mutations in the target genes with the level of significance set at *p* = 0.05.

## Results

A total of 266/526 (50.6%) stored DNA specimens were confirmed as *M. genitalium* positive using the Sacace *M. genitalium* Real-TM PCR assay (Table 1). These DNA specimens were obtained from 126 men and 140 women. The median age of all 266 participants was 27 years (IQR 24–32) [males: 28 years (IQR 24–33); females: 26 years (IQR 23–31)]. A significant difference in HIV serostatus was observed between males (57/126 HIV-infected; 45.2%) and females (90/140 HIV-infected; 64.3%) (*p* = 0.002) in the *M. genitalium*-infected cohort analysed*.*

Macrolide resistance-associated mutations (A2058G/A2059G) were not detected in any of the specimens analysed. Mutations in the QRDR of the *gyrA* and *parC* genes were detected in 9/266 (3.4%) and 54/266 (20.3%) specimens, respectively (Table 2), with no significant differences observed between males and females (*gyrA*: *p* = 0.88; *parC*: *p* = 0.62). HIV serostatus was not associated with the presence or absence of mutations in either *gyrA* (*p* = 0.99) or *parC* (*p* = 0.51).

**Table 2:** Fluoroquinolone resistance-associated mutations and corresponding amino acid changes in *Mycoplasma genitalium*, Gauteng Province, South Africa

Gene	Mutation/s	Amino acid change/s	No. of samples (*n* = 266)	Frequency (%)	Associated with clinically-relevant antimicrobial resistance	
					Yes/No/ Unknown	Reference
*gyrA*	G207A	M69I	2	0.8	Unknown	N/A
	G231A	No change	1	0.4	N/A	N/A
	A288G	No change	2	0.8	N/A	N/A
	A318G	No change	1	0.4	N/A	N/A
	C321T	No change	1	0.4	N/A	N/A
	T348C	No change	1	0.4	N/A	N/A
	A353G	H118R	1	0.4	Unknown	N/A
*parC*	C234T	No change	1	0.4	N/A	N/A
	G259 T	D87Y	1	0.4	Yes	[29, 35, 50, 52]
	G291A	No change	1	0.4	N/A	N/A
	A338G	D113G	1	0.4	Unknown	N/A
	C184T + C234T	P62S	50	18.8	Unknown	[35, 46, 54]
	C184T + C234T + A338G	P62S + D113G	1	0.4	Unknown	N/A

*N/A* Not applicable

QRDR *gyrA* mutations comprised two single nucleotide polymorphisms (SNPs) resulting in altered amino acids at positions 69 (M69I) and 118 (H118R). The clinical relevance of these SNPs is unknown. The M69I alteration was detected in two specimens (2/266; 0.8%) sampled in 2013 while the H118R alteration occurred only once (1/266; 0.4%) in a sample collected in 2007. An additional six specimens contained single synonymous substitutions at nucleotide positions 231 (G231A), 288 (A288G), 318 (A318G), 321 (C321T) and 348 (T348C), resulting in no amino acid changes.

Mutations in the QRDR of *parC* were more frequently observed and included amino acid alterations at positions 62 (P62S), 87 (D87Y) and 113 (D113G) (Table 2). The clinically significant D87Y alteration in ParC was observed only once in 2013 (1/266; 0.4%). The P62S was the most common alteration observed in ParC (50/266; 18.8%) and was always accompanied by a synonymous substitution at nucleotide position 234 (C234T). The P62S (C184T)/C234T combination was observed in each year of the survey (2007–2014). In one specimen collected in 2010, the P62S alteration (+C234T nucleotide substitution) was observed in combination with another amino acid alteration at position 113 (D113G) of ParC. One additional synonymous *parC* nucleotide substitution, G291A, was observed in a single specimen collected in 2012.

## Discussion

We report the baseline prevalence rates of macrolide and fluoroquinolone resistance-associated mutations in *M. genitalium* among symptomatic and asymptomatic patients in Johannesburg, South Africa (2007–2014). No macrolide resistance-associated mutations were found in the 23S rRNA genes of *M. genitalium* analysed in this study, and only one specimen (0.4%) contained a known fluoroquinolone resistance-associated mutation (G259 T) in the *parC* gene resulting in amino acid change D87Y.

*M. genitalium* is a known cause of non-gonococcal urethritis (NGU) worldwide but syndromic management of NGU is mainly focused on the successful treatment of *C. trachomatis* infections. Three antimicrobial classes, namely tetracyclines, macrolides and fluoroquinolones, have shown antimicrobial activity against mycoplasmas. In South Africa, prior to 2008, ciprofloxacin and doxycycline were the recommended syndromic treatment options for patients presenting with genital discharge syndromes but due to the rapid emergence of fluoroquinolone-resistant *N. gonorrhoeae* in the country, the treatment regimen was changed in 2008 to single-dose cefixime and doxycycline [27]. Doxycycline is very effective in clearing *C. trachomatis* infections but has a low eradication rate for *M. genitalium* infection. Given concerns over the subsequent emergence of cefixime-resistant *N. gonorrhoeae* isolates in South Africa, and in the face of already high-level tetracycline resistance among gonococci, the treatment guidelines were once again changed in 2015. A key change was to replace doxycycline with azithromycin, a potent macrolide with a long half-life and good tissue penetration [12, 31]. Single dose azithromycin (1 g) is an attractive treatment option for STI patients with *C. trachomatis* and, although not ideal, may also treat co-existing *M. genitalium* infections. Azithromycin has now been adopted in many countries as the first line treatment option for NGU [32]. The additional impact of azithromycin as a component of a dual treatment regimen for gonorrhoea makes this drug a rational choice for the syndromic management of male urethritis syndrome (MUS) and vaginal discharge syndrome (VDS) [33].

An increase in the prevalence of macrolide resistant *M. genitalium* strains has been reported in many parts of the world, signifying a strong association between the presence of specific mutations in the 23S rRNA gene of *M. genitalium* and macrolide treatment failure [15, 34–37]. It is now widely accepted that a 1 g single dose of azithromycin may be more likely to select for macrolide resistance as compared to extended azithromycin treatment (500 mg on day 1, 250 mg daily on days 2–5) although macrolide resistant *M. genitalium* infections are unlikely to respond to either regimen [38, 39]. The widespread use of 1 g azithromycin in countries such as Australia, Canada, Greenland, the United Kingdom and Japan has caused rapid selection of resistant mutants, resulting in the detection of macrolide resistance-associated mutations in 31–100% of *M. genitalium* analysed [36, 40–43]. The rise in macrolide-resistant *M. genitalium* infections has heralded calls to use an extended azithromycin regimen or revert to doxycycline as first-line treatment of NGU. Moxifloxacin is recommended in settings where macrolide resistance is already well established [32].

Limited information is available on the prevalence of macrolide resistance-associated mutations in South Africa with only two studies reporting prevalence rates of mutations in the 23S rRNA gene of *M. genitalium* [44, 45]. The first study showed a 9.8% prevalence of the A2058G 23S rRNA mutation in 41 *M. genitalium*-positive specimens collected between 2011 and 2012 in the Mopani District, Limpopo Province [45]. These remnant specimens were obtained from sexually active females aged 18–49 years who attended primary health care clinics and participated in a cross-sectional study of vaginal, rectal and oral gonococcal and chlamydial infections [45]. The second study investigated macrolide resistance-associated mutations in the 23S rRNA genes of *M. genitalium* detected in females attending a pregnancy termination clinic at the George Mukhari Academic Hospital in Pretoria in 2012 and 2016 [44]. No macrolide resistance-associated mutations were detected in the 2012 specimens but 2/8 (25.0%) of the *M. genitalium*-positive 2016 specimens contained the A2059G 23S rRNA mutation [44]. Macrolide resistance-associated mutations were not detected in any of the *M. genitalium*-positive specimens analysed in our study but could emerge due to the recent incorporation of azithromycin into the 2015 South African national STI treatment guidelines.

Fluoroquinolones are often recommended as a second-line treatment option for *M. genitalium* infection but vary in terms of efficacy and in vitro activity against the organism [46]. Fluoroquinolones that are highly active against *M. genitalium* include moxifloxacin, gatifloxacin, sitafloxacin and some of the newer fluoroquinolones, while ciprofloxacin, ofloxacin and levofloxacin are less efficacious for the treatment of *M. genitalium* infections [17, 47, 48]. Moxifloxacin is the treatment of choice for macrolide-resistant *M. genitalium* infections. However, recent reports of moxifloxacin treatment failure with reduced cure rates of macrolide-resistant *M. genitalium* infections in the Asia-Pacific region have emerged; these are all linked to the presence of fluoroquinolone resistance-associated mutations in *gyrA* and *parC* [20]. A number of these resistance-associated mutations in *gyrA* and *parC* have been described [35, 46, 49–51]. Our findings mirror those from other published studies, whereby the frequency of mutations observed in *parC* was higher than in *gyrA* (19.5% v 1.1%) [35, 46, 49, 50]. Only two amino acid alterations were detected in GyrA (M69I in 2 specimens and H118R in one specimen) but none of these alterations have previously been associated with fluoroquinolone resistance.

The most commonly reported amino acid alterations associated with fluoroquinolone resistance are found at positions 83 and 87 of ParC (corresponding to positions 80 and 84 in *E. coli*) [35, 46, 49–51]. The amino acid change D87Y in ParC has been described in specimens from Japan, France, Russia, Estonia and Australia and was found in only one specimen from this study [35, 49–52]. The newly described D113G alteration in ParC was detected in one specimen but it is not known if this alteration contributes to fluoroquinolone resistance. The most prevalent amino acid alteration in ParC from this study was P62S (18.8%). The P62S ParC alteration, caused by the missense mutation C184T, has been described previously in a whole genome sequenced *M. genitalium* strain (M6282) that originated from a male with non-chlamydial NGU in Miyazaki, Japan [53]. The M6282 strain contained both the C184T missense mutation as well as a synonymous C234T mutation the QRDR of *parC*, however this strain has demonstrated in vitro phenotypic susceptibility to fluoroquinolones [15]. The P62S alteration was detected in 9.3% of *M. genitalium*-positive individuals in Melbourne, Australia and in 14.8% of *M. genitalium*-positive men-who-have-sex-with-men (MSM) in Alabama, USA [46, 54]. The location of the P62S alteration in the QRDR of *parC* indicates that it may play a role in the evolution of fluoroquinolone resistance but current evidence suggests it does not confer phenotypic resistance [15]. STI patients in Johannesburg could have been exposed to circulating fluoroquinolone resistant *M. genitalium* strains in the community, and this could explain the presence of QRDR mutations observed in *M. genitalium* from this study. They may also have been exposed to quinolones used as treatment for other indications such as urinary tract infections. Moxifloxacin is currently one of five second-line drugs used to treat multi-drug resistant *Mycobacterium turberculosis* (TB) in South Africa, but due to concerns of widespread unrestricted use in primary healthcare centres and consequently the development of moxifloxacin resistance in TB, it is unlikely to be included in future national STI treatment guidelines.

Limitations of the study included the inability to determine the association between the presence of amino acid alterations in the QRDR of *gyrA* and *parC* detected in this study and patient treatment outcomes. Patients presenting with genital discharge at primary health care clinics in South Africa are treated syndromically and they might not return to the same clinic for follow-up assessment and treatment if initial treatment failed to cure their infection. Antimicrobial susceptibility assays for *M. genitalium* are also rarely performed due to the technical difficulties in culturing *M. genitalium* from clinical specimens, and therefore attempts to associate phenotypic resistance with genotypic findings are lacking [26]. Another limitation was the inability to confirm all *M. genitalium*-positive specimens that were stored at -70 °C. Unsuccessful amplifications were mostly observed in older specimens and may have been due to DNA degradation during storage or due to low bacterial loads of *M. genitalium* in these specimens. Carlson and Jensen (2010) demonstrated that freezing of *M. genitalium*-positive clinical specimens, as well as extracted DNA, leads to a significantly lower *M. genitalium* DNA load and a decreased sensitivity to that obtained by testing fresh specimens, especially in those with an initial low DNA load [55]. Finally, the samples tested came from one city in South Africa and may not be geographically representative at national level.

## Conclusions

In conclusion, we report the absence of macrolide resistance-associated mutations in *M. genitalium*-positive patients presenting with urogenital discharge to the Alexandra Health Centre between 2007 and 2014 and among asymptomatic *M. genitalium*-positive, HIV-positive individuals attending an urban HIV outpatient clinic in Johannesburg in 2007. The low number of known fluoroquinolone resistance-associated mutations observed in the QRDR of *M. genitalium* in this study could strengthen the case for reverting to this class of antibiotic if macrolide resistance become widespread in South Africa. However, resistance to macrolides could emerge due to the incorporation of azithromycin in the 2015 South African national STI treatment guidelines. Countries with high-level macrolide resistance in *M. genitalium* could implement rapid *M. genitalium* and macrolide resistance testing at point-of-care, using commercial assays such as the SpeeDX Resistance Plus™ MG test to facilitate appropriate targeted treatment [56]. Ongoing surveillance of macrolide resistance-associated mutations in *M. genitalium* positive cases is warranted and will be performed in our setting to support the continued use of azithromycin, which is included in the current national STI syndromic management guidelines.

## Abbreviations

AIDS: Acquired immune deficiency syndrome
bp: Base pair
CHIVSTI: Centre for HIV and Sexually Transmitted Infections
DNA: Deoxyribonucleic acid
HIV: Human immunodeficiency virus
IQR: Interquartile range
MSM: Men-who-have-sex-with-men
MUS: Male urethritis syndrome
NGU: Non-gonococcal urethritis
NICD: National Institute for Communicable Diseases
NMS: National Microbiological Surveillance
PCR: Polymerase chain reaction
PID: Pelvic inflammatory disease
PLWHA: People living with HIV/AIDS
QRDR: Quinolone resistance-determining region
rRNA: Ribosomal ribonucleic acid
STI: Sexually transmitted infection
TB: *Mycobacterium tuberculosis*
VDS: Vaginal discharge syndrome
